# Zweifach–Fung Microfluidic Device for Efficient Microparticle Separation: Cost-Effective Fabrication Using CO_2_ Laser-Ablated PMMA

**DOI:** 10.3390/mi15070932

**Published:** 2024-07-22

**Authors:** Cristian F. Rodríguez, Mateo Báez-Suárez, Carolina Muñoz-Camargo, Luis H. Reyes, Johann F. Osma, Juan C. Cruz

**Affiliations:** 1 Department of Biomedical Engineering, Universidad de los Andes, Cra. 1E No. 19a-40, Bogotá 111711, Colombia; cf.rodriguez@uniandes.edu.co (C.F.R.); m.baezs@uniandes.edu.co (M.B.-S.); c.munoz2016@uniandes.edu.co (C.M.-C.); jf.osma43@uniandes.edu.co (J.F.O.); 2 Neuroscience Group of Antioquia, Cellular and Molecular Neurobiology Area, School of Medicine, SIU, University of Antioquia, Cl. 62 No. 52-59, Medellin 050010, Colombia; 3 Grupo de Diseño de Productos y Procesos (GDPP), Department of Chemical Engineering, Universidad de los Andes, Cra. 1E No. 19a-40, Bogotá 111711, Colombia; lh.reyes@uniandes.edu.co; 4 Department of Electrical and Electronic Engineering, Universidad de los Andes, Cra. 1E No. 19a-40, Bogotá 111711, Colombia

**Keywords:** labs-on-a-chip, microfluidic-device, microparticle-separator, COMSOL, low-cost, SWOT

## Abstract

Microfluidic separators play a pivotal role in the biomedical and chemical industries by enabling precise fluid manipulations. Traditional fabrication of these devices typically requires costly cleanroom facilities, which limits their broader application. This study introduces a novel microfluidic device that leverages the passive Zweifach–Fung principle to overcome these financial barriers. Through Lagrangian computational simulations, we optimized an eleven-channel Zweifach–Fung configuration that achieved a perfect 100% recall rate for particles following a specified normal distribution. Experimental evaluations determined 2 mL/h as the optimal total flow rate (TFR), under which the device showcased exceptional performance enhancements in precision and recall for micrometer-sized particles, achieving an overall accuracy of 94% ± 3%. Fabricated using a cost-effective, non-cleanroom method, this approach represents a significant shift from conventional practices, dramatically reducing production costs while maintaining high operational efficacy. The cost of each chip is less than USD 0.90 cents and the manufacturing process takes only 15 min. The development of this device not only makes microfluidic technology more accessible but also sets a new standard for future advancements in the field.

## 1. Introduction

Microfluidic devices, commonly known as Labs-on-a-Chip (LOCs), are transforming our ability to manipulate fluids at the microscale [1,2,3]. These devices offer substantial benefits across various fields, significantly advancing experimental methodologies by reducing the volume of chemicals and materials needed, thereby promoting sustainability and efficiency [4,5]. Enhanced heat and mass transfer rates at the microscale enable quicker experimental processes, boosting overall experimental throughput [6,7]. Moreover, microfluidics maintains steadier conditions during operations, ensuring that results are both reliable and reproducible, contrasting favorably with traditional methods [8,9].

LOCs have become indispensable in industries such as chemical production and pharmaceuticals due to their capacity for executing complex processes [10,11,12]. Within the confines of these microscale systems, it is possible to conduct a variety of processes with unprecedented precision and efficiency [13,14]. These processes encompass the synthesis, analysis, separation, and purification of materials, ranging from nano to micrometer scales [15,16,17,18]. The ability to perform such intricate operations on a microscale not only enhances the quality and specificity of the outcomes but also significantly reduces the time and resources required for their completion. Such efficiency and versatility underline the growing interest in LOC technology for developing innovative solutions in chemical synthesis, pharmaceutical research, and the production of highly purified materials [19].

In the field of microfluidics, devices for the purification of materials at nano and micro scales have evolved into two broad categories: active and passive systems. Active microfluidic separators utilize external forces such as electrical, magnetic, or acoustic energies to manipulate and sort the particles within microchannels [20,21,22,23]. These techniques offer precise control, allowing for the separation based on specific physical properties of the particles.

Conversely, passive microfluidic separators leverage the inherent physical properties of fluids and particles, alongside sophisticated channel designs, to achieve separation. These methods harness intrinsic physical forces—gravity, diffusion, inertia, and hydrodynamics—without the need for external energy sources, making the precise control of channel geometry a critical factor for efficient separation [24,25,26,27].

A notable passive separation technique in microfluidics is the Zweifach–Fung effect, first described by Y.C. Fung and B.W. Zweifach in their foundational study “Microcirculation: mechanics of blood flow in capillaries” at the University of California [28]. They meticulously documented the dynamic behavior of particulate suspensions at asymmetric bifurcations within microchannels. Their observations revealed a distinct divergence in the volume fraction of particles at these points, leading to unequal distributions among the daughter branches.

Their research detailed how particle suspensions behave differently at asymmetric bifurcations within microchannels, leading to varied particle distribution across the branches, a principle crucial for precise particle sorting [29]. This mechanism has been integrated into the design of microfluidic devices that aim to achieve exact particle distribution, essential for the functionalities of LOC applications [30].

Traditional methods for manufacturing microfluidic devices typically require cleanroom facilities. However, in recent decades, there has been a notable shift towards developing novel, cost-effective approaches that do not rely on a cleanroom environment [31,32,33,34]. One prominent approach involves the use of thermoplastics, which have gained increasing popularity in the field due to their numerous advantages [35,36].

Thermoplastics offer several benefits, including faster production times and lower costs, making them highly suitable for scaling up the manufacturing process of microfluidic devices [37,38]. Among the various thermoplastics that have garnered significant interest in recent years are Cyclic Olefin Copolymers (COCs), Polypropylene (PP), Polystyrene (PS), Polycarbonate (PC), Polyethylene terephthalate (PET), Polyimide (PI), and Poly (methyl methacrylate) (PMMA) [39,40,41,42,43,44,45].

PMMA, in particular, has attracted increasing attention, for its unique properties, such as high transparency, biocompatibility, and easy manipulation [46,47,48,49]. This surge in interest can be attributed, in part, to the work reported by Klank et al. in 2002, which studied the manufacture of microfluidic PMMA using CO_2_-laser micromachining as a rapid and cost-effective alternative for manufacturing microfluidic devices [49]. Since the publication of the article by Klank et al., various microfluidics devices have been manufactured using the CO_2_-laser micromachining technique [50,51]. These devices have been applied in diverse areas such as droplet generation, micromixing, and micro-separation [52,53,54,55,56]. Specifically, different microfluidic separators have been developed using principles such as inertial separation, pinched flow fractionation, and deterministic lateral displacement.

However, there are a few instances of microfluidic devices employing the Zweifach–Fung effect being manufactured using laser ablation in PMMA, and in particular those with channels smaller than 100 µm [57,58]. Given the broad application potential of laser ablation in microfluidics, there is significant interest in exploring the feasibility of using this technique to fabricate devices based on the Zweifach–Fung effect, especially for microparticle separation [59,60,61].

To address this challenge, we present the development of a passive microfluidic device based on the Zweifach–Fung principle, fabricated using an economical laser ablation technique in polymethyl methacrylate (PMMA), thereby circumventing the need for a cleanroom. By demonstrating the feasibility of this approach, we aim to reduce economic barriers and broaden the accessibility and application of microfluidic devices, fostering greater innovation in the field.

## 2. Materials and Methods

### 2.1. Materials

Hexane (99%), Glutaraldehyde (25%), Tween 80, and mineral oil were purchased from Sigma–Aldrich (St. Louis, MO, USA). Polymethyl methacrylate (PMMA) sheets of 2 mm and 4 mm thickness were purchased from local distributors (Bogotá, Colombia).

### 2.2. Computational Modeling of Microparticle Separation Dynamics

The dynamics of microparticle separation within microfluidic devices was analyzed using a Lagrangian approach in COMSOL Multiphysics 6.2 software (COMSOL Inc., Stockholm, Sweden). This method, known as particle tracing, allowed for an analysis of the effect of the flow and the magnetic field on the particle trajectories.

The flow behavior was modeled based on the fundamental principles of fluid dynamics under laminar flow conditions, governed by the Navier–Stokes equations for momentum conservation and the continuity equation for mass conservation [62,63]. These are represented as Equation (1) for the Navier–Stokes equations and Equation (2) for the continuity equation.

(1) $$\rho \left(u\cdot \nabla \right)u=\nabla \cdot \left[-pI+\mu \left(\nabla u+{\left(\nabla u\right)}^{T}\right)\right]+F$$

(2) ρ∇⋅u=0

where ρ is the fluid density, *u* is the fluid velocity, *p* is the fluid pressure, I is the identity matrix, and *F* is the external forces. The particle tracking was performed using the Lagrangian approximation. This method relies on the assumption that particle motion can be described by Newton’s second law, which is encapsulated in Equation (3). This equation provides a framework for analyzing how forces affect particle trajectories in microfluidic devices.

(3) $$Ft=\frac{d\left(m_{p}*v\right)}{dt}$$

where Ft is the sum of all forces acting on the particles, v is the particle velocity, and $m_{p}$ is the particle mass. The influence of the fluid on the particles within the microfluidic device was quantitatively modeled by incorporating the drag force, as dictated by Stokes’ Drag Law. This fundamental force is described by Equation (4).

(4) $$Fd=\left(u-v\right)*\frac{m_{p}}{\tau_{p}}$$

where *u* is the velocity field, v is the particle velocity, $m_{p}$ is the particle mass, and $\tau_{p}$ is the particle velocity response time or Lagrangian time scale (Equation (5)).

(5) $$\tau_{p}=\frac{p_{p}*{d_{p}}^{2}}{18\mu }$$

where *μ* is the viscosity, $p_{p}$ is the particle density, and $d_{p}$ is the particle diameter.

### 2.3. Simulation Specifics and Particle Properties

Simulations were meticulously crafted based on the unique properties of the particles used, including densities and diameters of polystyrene and chitosan. This approach ensured precise and relevant analysis for each test scenario.

These properties were modeled using a normal distribution probability density function, represented by Equation (6), and the simulations were carried out over 25 iterations.

(6) $$f\left(d_{p}\right)=\frac{1}{\sigma \sqrt{2\pi }}{\mathrm{e}}^{-\frac{1}{2}{\left(\frac{d_{p}-\mu }{\sigma }\right)}^{2}}$$

where $d_{p}$ is the diameter of the particle, μ is the mean diameter of the particle, and σ is the standard deviation. The specifics of the means and standard deviations are detailed in Table S1. During the simulations, particles were introduced into the system at a consistent rate of 100 every 0.1 s, ensuring uniform conditions for analysis.

For chitosan microparticles, the size distribution was aligned with the results from our experimental tests, as previously documented [64]. This alignment was intended to maintain the consistency of evaluation metrics used in earlier studies, thereby ensuring comparability and methodological continuity.

### 2.4. Discretization and Solvers

**Figure 1:**
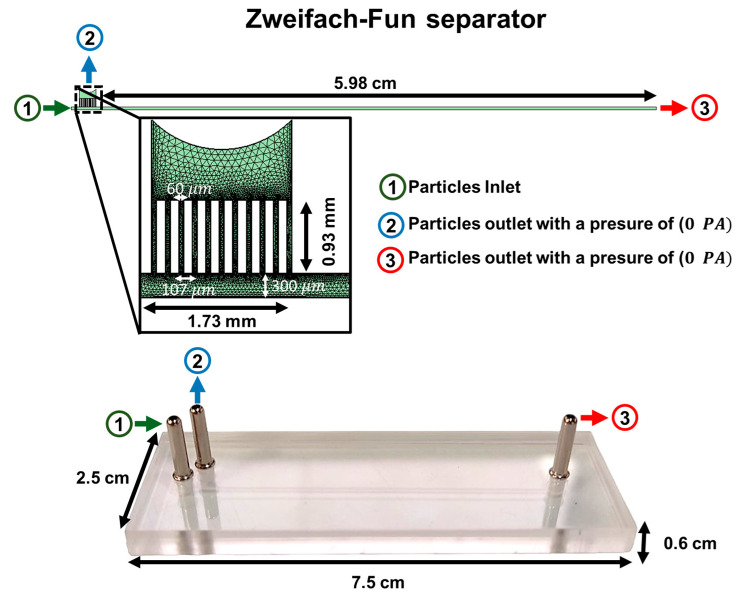
Meshing and boundary conditions for simulation setups for the eleven-channel Zweifach–Fung device. The microparticle inlet is indicated in green and labeled ‘1’, where particles are introduced at flow rates of 2, 20, or 200 mL/h. The outlet for particles smaller than 40 µm is marked in blue and labeled ‘2’, while the outlet for particles larger than 40 µm is highlighted in red and labeled ‘3’.

The computational domain within the microfluidic devices was discretized using triangular elements. The Zweifach–Fung passive separator was modeled using 23,573 domain elements and 2946 boundary elements. This level of discretization ensures mesh convergence, as presented in Figure S1. A comprehensive breakdown of the discretization for additional geometries that were simulated but not manufactured, along with the parameters used for the simulations, is available in Table S2. Moreover, Figure 1 (upper panel) displays the mesh configurations and boundary conditions applied in these simulations, particularly focusing on the devices that were manufactured. Figure 1, lower panel, shows an actual picture of the manufactured device before it was glued, while Figure S2 depicts the device after the gluing process.

The computational approach to solving the equations for microfluidic systems was carried out in a stepwise manner. Initially, the analysis began with a fully coupled study using the MUMPS (MUltifrontal Massively Parallel sparse direct Solver) [65,66,67]. This solver, which employs Gaussian factorization through a multifrontal method, is particularly adept at handling complex, coupled systems.

Upon the successful completion of the coupled study, the data generated from the CFD were integrated into the Lagrangian particle tracking model. This next phase utilized the Generalized Minimum RESidual (GMRES) solver. This iterative solver is optimal for handling the large-scale simulations required by our study, reducing memory usage and enhancing computational efficiency. The GMRES solver enhances the modeling process by iteratively constructing a Krylov subspace from the residuals of the linear system equations.

### 2.5. Low-Cost Manufacture

**Figure 2:**
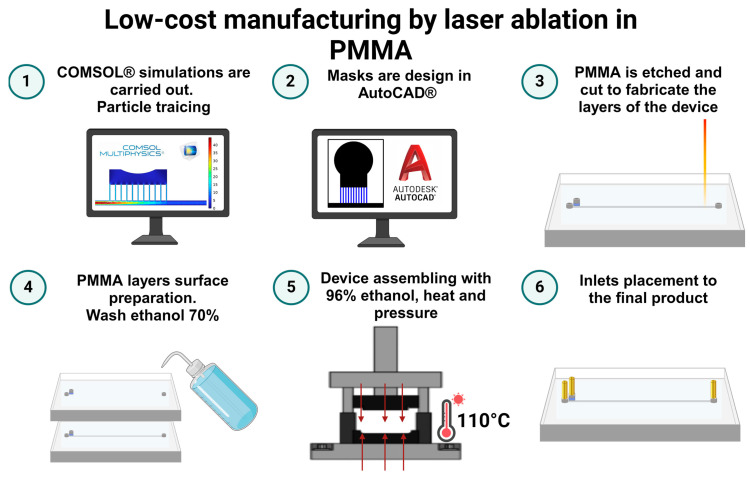
Manufacturing process of magnetic and Zweifach–Fung microfluidic devices via laser ablation in PMMA. The procedure begins with (1) computational design and simulation in COMSOL Multiphysics 6.2 software (COMSOL Inc., Stockholm, Sweden) to determine optimal geometry. The design is then (2) refined in AutoCAD, where engraving and cutting paths are precisely demarcated. (3) The PMMA sheets are accurately cut and engraved using a TROTEC^®^ laser system (TROTEC, Marchtrenk, Austria) following the color-coded design specifications. (4) Post-ablation, the sheets are cleansed with ethanol to prepare the surfaces for bonding. (5) Sheets are fused under pressure and heat to form the microfluidic structure. (6) Inlets and outlets are integrated to finalize the device, making it ready for experimental application.

The microfluidic separator device was fabricated using an economical laser ablation method on polymethyl methacrylate (PMMA) substrates, as previously reported by Ortegon et al. [68]. Figure 2 shows graphically the manufacturing process.

The design phase involved detailed simulations using COMSOL Multiphysics 6.2 software (COMSOL Inc., Stockholm, Sweden), essential for defining the device’s geometry. This geometry was then exported to AutoCAD (AutoDesk Inc., Mill Valley, CA, USA), where the design was refined by coloring the engraving areas in black and blue and the cutting zones in red to ensure precision during the ablation process.

Following this, the PMMA sheets were processed using a TROTEC^®^ laser cutting system (Marchtrenk, Austria). The system was set to a power of 100 and a speed of 0.4 for cutting, and a power of 15 with a speed of 12 for engraving. Afterward, the PMMA sheets underwent a thorough cleaning with a 70% ethanolic solution to remove any residual debris, ensuring a pristine surface for bonding.

The clean sheets were then bonded using a thin application of 96% ethanol and subjected to constant pressure at 110 °C for five minutes. This step was critical to ensure a strong, durable bond between the PMMA layers. The final assembly step involved accurately attaching inlets and outlets to the microfluidic device, thus equipping it for upcoming experimental applications. Figure S2 shows close-up images of the fabricated device after gluing, and Table 1 show the materials cost used for the manufacture of the microfluidic device.

### 2.6. Particle Synthesis for Assessing Device Performance

To comprehensively evaluate the performance of microfluidic separators, we synthesized chitosan microparticles and used commercial polystyrene particles (MV-F02, Microvec, Pińczów, Poland).

#### Microparticles of Chitosan

Chitosan microparticles were synthesized using a modified version of the protocol described by Rodríguez et al. [64]. Initially, the aqueous phase was prepared by dissolving 2% (*w*/*v*) chitosan in a 4% (*v*/*v*) acetic acid solution, stirred magnetically at 500 RPM for 24 h to ensure complete dissolution. Concurrently, the oil phase, composed of Tween 80 and mineral oil, was mixed to achieve a 2% (*v*/*v*) concentration.

After the preparation of both phases, 5 mL of the aqueous chitosan solution was gradually added to 100 mL of the oil phase. The mixture was then stirred at 600 RPM for 10 min using a Hei-TORQUE Precision 200 (Heidolph, Schwabach, Germany) mechanical stirrer to create a water-in-oil (W/O) emulsion. Following the emulsion formation, 1 mL of glutaraldehyde solution was added, and stirring continued at 300 RPM for an additional 2 h to promote cross-linking within the microparticles.

The chitosan microparticles were then isolated by centrifugation at 3600 RPM for 10 min and washed three times, first with hexane and subsequently with Type II water, to remove residual chemicals.

### 2.7. Experimental Separation Tests

#### 2.7.1. Sensitivity Evaluation of the Passive Zweifach–Fung Microfluidic Devices

The sensitivity of the Passive Zweifach–Fung microfluidic device was assessed by evaluating its ability to correctly route commercial polystyrene microparticles labeled with rhodamine (B MV-F02, Microvec, Pińczów, Poland). Given that all microparticles involved in the test were smaller than 40 µm, they were expected to be channeled exclusively to outlet 1, following the device’s design specifications.

During testing, microparticle solutions were introduced into the microfluidic device at total flow rates (TFRs) of 2, 20, and 200 mL/h using a KDS-100 syringe infusion pump from W.P. Instruments (Holliston, MA, USA) The experimental setup, including the infusion pump, is illustrated in Figure S3. The separation process was monitored, and microparticles were collected from two distinct outlets of the device—outlet 1 and outlet 2.

Each sample collected was analyzed using a spectrofluorometer (0239D-2219 FluoroMax plus C, Horiba, Kyoto, Japan) with the excitation wavelength set at 545 nm and emission measured at 560 nm. This facilitated quantification of microparticles from each outlet, allowing for an accurate assessment of the device’s separation capabilities.

To analyze the results, particles detected in outlet 1 were expected, and their presence confirmed the device’s functionality. Conversely, any microparticles found in outlet 2 were categorized as false negatives, indicating a failure in the device’s ability to correctly separate and route particles smaller than 40 µm. The recall or sensitivity was calculated as the ratio of microparticles smaller than 40 µm correctly identified in outlet 1 compared to those smaller than 40 µm and collected in outlet 2. The separation performance was evaluated for 5 microfluidic chips to analyze the deviation and possible variations associated with the manufacturing technique.

#### 2.7.2. Passive Chitosan Microparticle Separation

Performance comparison of the Passive Zweifach–Fung microfluidic against our previously utilized passive separation device was conducted using a standardized chitosan microparticle separation test as described in our earlier publication [64].

**Figure 3:**
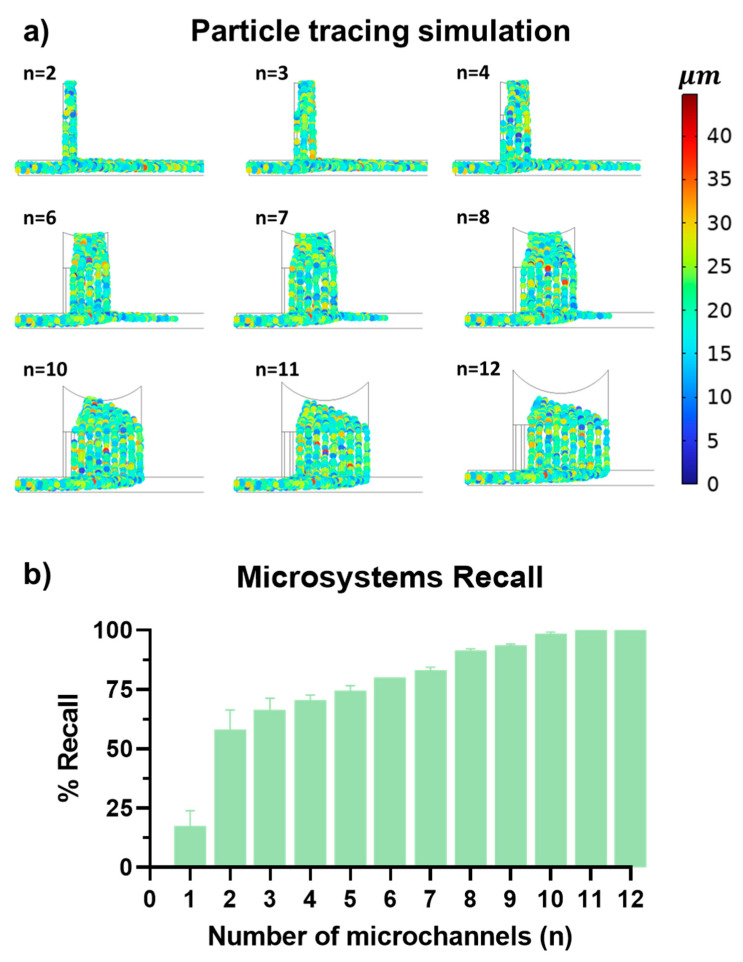
In silico evaluation of the recall efficiency in Zweifach–Fung microfluidic devices with multi-channels. (**a**) Particle trajectories: Color-coded simulation imagery depicting particle trajectories within microchannels. Colors represent particle sizes: blue for 5 μm, green for 20 μm (most prevalent), and red for 40 μm. This illustrates variations in flow dynamics and separation efficiency across different channel configurations. (**b**) Recall efficiency plot: Column plot displaying recall efficiency across varying numbers of microchannels from 1 to 12. A marked increase in efficiency is observed as the number of channels rises, with the configuration comprising 11 channels demonstrating the highest recall rate, identifying this as the optimal channel number.

Initially, we introduced a solution containing chitosan microparticles into the Zweifach–Fung type passive separation device using a KDS-100 syringe infusion pump from W.P. Instruments (Holliston, MA, USA). The operational parameters, including the flow rate, were optimized based on the findings from experiments and simulations detailed in Figure 3. These results indicated that a flow rate of 2 mL/h is optimal for effective particle separation.

Samples of 1 mL were collected from each of the device’s outlets and analyzed under an optical microscope (Primo Star, ZEISS, Jena, Germany) equipped with Zen 3.7^®^ software (ZEISS, Jena, Germany). The analysis focused on determining the size distribution of the particles from each outlet.

Separation efficiency was evaluated as a binary separation task, with particles smaller than 40 µm classified as positive outcomes and those larger than 40 µm as negative outcomes. This binary approach provided a clear, quantifiable measure of the Zweifach–Fung device’s capabilities relative to our previous model, evaluating the parameters recall, precision, F1 score, and accuracy. The separation performance was evaluated for 5 microfluidic chips to analyze the deviation and possible variations associated with the manufacturing technique.

Recall was calculated as the ratio of true positives (particles smaller than 40 µm correctly identified in outlet 1) to the sum of true positives and false negatives (particles smaller than 40 µm incorrectly identified in outlet 2). This measure indicates the device’s ability to separate all relevant microparticles accurately.

Precision was determined as the ratio of true positives to the sum of true positives and false positives (particles larger than 40 µm incorrectly identified in outlet 1). This metric reflects the accuracy of the device in separating only the relevant microparticles without misclassifying larger particles.

F1 score, the harmonic mean of precision and recall, was used to provide a single measure of the device’s performance, balancing both false positives and false negatives. This score offers a comprehensive view of the device’s effectiveness in particle separation.

Accuracy was calculated as the ratio of correctly identified particles (both true positives and true negatives) to the total number of particles. This measure encompasses both its ability to correctly identify smaller particles and exclude larger ones.

## 3. Results and Discussion

### 3.1. Geometric Optimization of Passive Microfluidic Devices

The effectiveness of passive microfluidic systems is greatly influenced by flow dynamics, which are determined by the device geometries. In this study, we focused our investigation on optimizing the number of channels in Zweifach–Fung systems. This key design parameter significantly impacts the fluid dynamics and the efficiency of particle separation.

The computational study, leveraging a Lagrangian model within COMSOL Multiphysics 6.2 software (COMSOL Inc., Stockholm, Sweden), aimed to pinpoint the optimal number of microchannels for the efficient separation of particles averaging of 20 μm in size. These simulations depicted particle trajectories within a variety of microchannel arrangements and enabled a parametric analysis to quantify the system’s recall ability.

As depicted in Figure 3, part (a) shows simulated particle trajectories color-coded by size: blue for approximately 5 μm, green for 20 μm, and red for 40 μm. This color-coding clarifies how different-sized particles navigate the microchannels, providing insights into the system’s separation efficiency. Part (b) of Figure 3 displays a column graph illustrating the recall performance for configurations ranging from one to 12 microchannels. A clear trend emerges, showing that increasing the number of microchannels enhances recall efficiency, with an 11-channel design achieving a perfect recall rate of 100%.

Initial findings revealed that a single channel configuration resulted in a recall rate below 25%, setting a performance baseline. The introduction of additional channels markedly improved this rate, attributed to reduced fluid resistance within the microchannels compared to the main channel. Despite its wider cross-section, the main channel’s extended length increases fluid resistance, hindering particle flow. By increasing the number of microchannels, we effectively distributed the flow and minimized overall resistance, enhancing the lateral forces that direct particles toward the outlet more efficiently.

The 11-channel configuration, achieving the highest recall rate, was selected as the optimal design. This configuration was fabricated and subjected to extensive experimental testing and additional simulations to thoroughly understand its performance dynamics and operational characteristics under varying conditions. By leveraging the potential of employed multiphysics simulations, it was possible to reduce the extent of experimental testing, thereby lowering the experimental time [69,70].

### 3.2. Intrinsic Errors and Dimensional Reductions in PMMA Microfluidic Devices Fabricated by Laser Ablation

**Figure 4:**
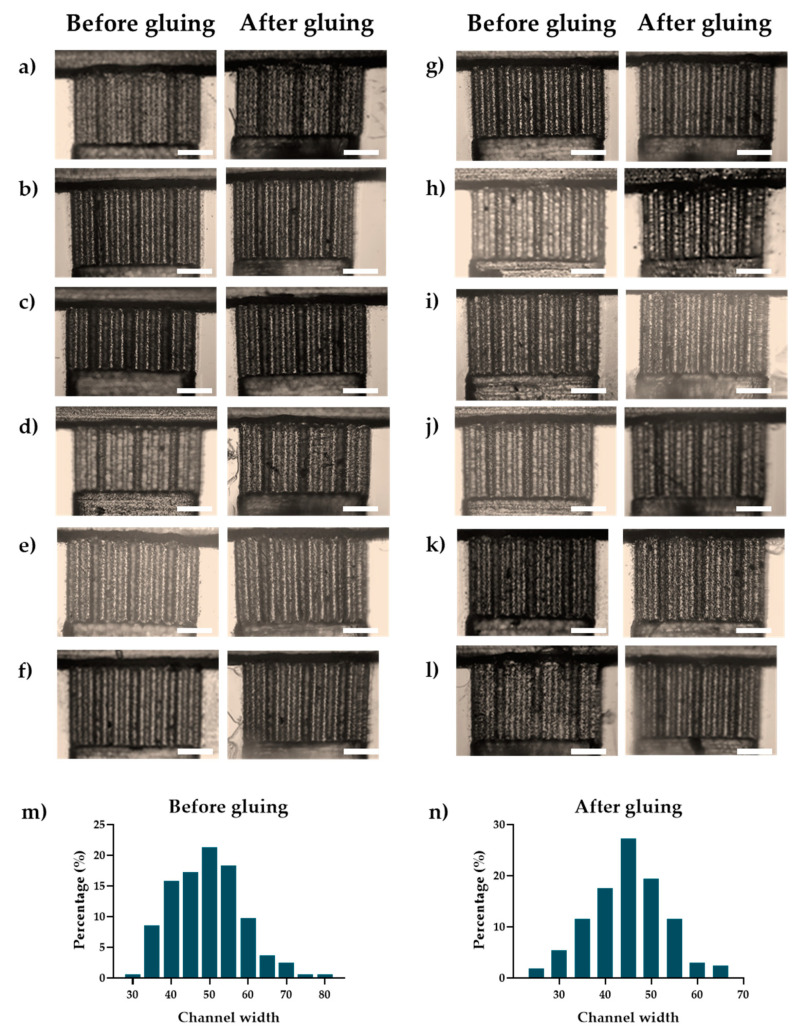
Microchannel before and after gluing. (**a**–**l**) Image collected by optical microscope before and after gluing with a 4× objective. Scale bar 500 μm. (**m**) Channel width histogram before gluing. (**n**) Channel width histogram after gluing.

The low-cost manufacturing technique of laser ablation in PMMA inherently introduces errors, as documented in the existing literature [48]. Figure 4 presents a comparative analysis of the widths of 12 microfluidic channels measured both before and after the bonding process. Initially, these channels exhibit a mean width of 49.40 μm ± 9.16 μm. Following the bonding process, the mean width decreases to 44.85 μm ± 8.27 μm, representing an approximate reduction of 5 μm. This reduction is also accompanied by a noticeable decrease in standard deviation. The elimination of channels exceeding 65 μm in width and the significant reduction in the occurrence of channels wider than 50 μm post-bonding, highlight the bonding process’s impact on channel geometry.

This observed narrowing effect is attributed to two primary factors: the Gaussian profile intrinsic to the laser ablation process, which contributes to initial dimensional variability, and the subsequent constriction during bonding, wherein ethanol-induced softening of the PMMA combined with applied compressive forces results in a consistent reduction in channel width [48]. These findings suggest that the bonding process not only reduces channel width but also enhances uniformity. This has significant implications for the performance of microfluidic devices, particularly in the context of particle separation, where diminished channel dimensions may restrict the range of particle sizes that can be effectively processed. Subsequent sections will delve into the implications of these dimensional changes for the operational capabilities and efficiency of microfluidic chips.

### 3.3. TFR Impact on Zweifach–Fung (ZF) Microfluidic Device Performance

**Figure 5:**
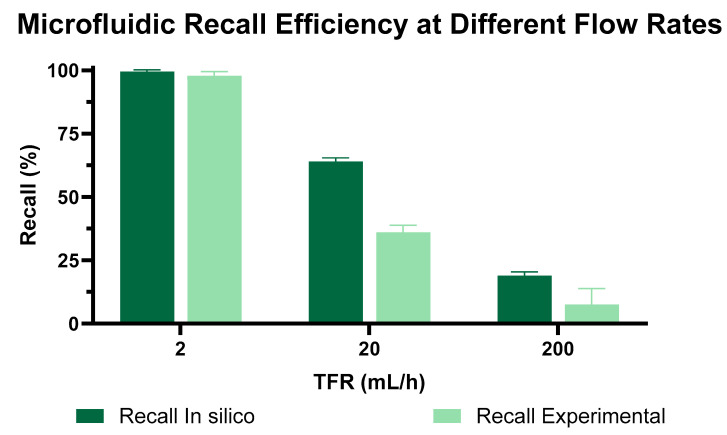
Comparison of recall efficiency between simulated and experimental results at varying TFRs: This section compares recall efficiencies as influenced by different total flow rates (TFRs), highlighting the impact of flow rate on separation performance and the discrepancies between simulated and actual experimental outcomes.

Following the geometric optimization of the Zweifach–Fung (ZF) microfluidic device, we evaluated its separation efficiency under various operational conditions. We utilized commercially available microparticles labeled with Rhodamine B to test the device’s performance, systematically varying the total flow rate (TFR) at 2, 20, and 200 mL/h to examine its impact on particle separation efficacy. The comparative results from both in silico predictions and experimental observations are compiled in Figure 5.

The data indicated that increasing TFRs detrimentally affected the separation efficiency. Both computational and experimental results confirmed this trend: higher TFRs led to more particles bypassing the intended bifurcations for separation. At the lowest TFR of 2 mL/h, the device demonstrated peak recall efficiency, achieving 100% recall. However, increasing the TFR to 20 mL/h resulted in a significant decrease in efficiency—experimental studies reported a recall rate of 45%, while simulations suggested a slightly higher rate of 65%. This disparity highlights the limitations of the computational model, which may not fully capture complex particle dynamics or subtle manufacturing nuances that become evident at higher flow rates.

At the highest TFR of 200 mL/h, efficiency plummeted to only 10% recall. This substantial decrease is attributed to the overwhelming influence of inertial forces at high fluid velocities, which override the device’s designed lateral forces that are crucial for size-based particle segregation. Consequently, the increased fluid velocity reduces the interaction time within the separation zones, diminishing the sorting mechanism’s effectiveness.

These findings, summarized in Figure 5, validate the importance of maintaining lower flow rates to achieve efficient particle separation within the ZF microfluidic design. The optimal flow rate of 2 mL/h was selected for subsequent device characterizations to ensure the most effective performance in practical applications. The deviations identified in the tests were 1.17 for the 2 mL/h test, 2.01 for the 20 mL/h test, and 4.48 for the 200 mL/h test. These deviations are attributed to the manufacturing technique. As discussed in Section 3.1, this technique has an inherent error that causes variations in the microchannel dimensions.

### 3.4. Chitosan Particle Separation

Building upon the established optimal flow parameters, our study extended into a more sophisticated examination of the microfluidic device’s capability, focusing not only on particle recall but also on precise separation and overall system accuracy.

**Figure 6:**
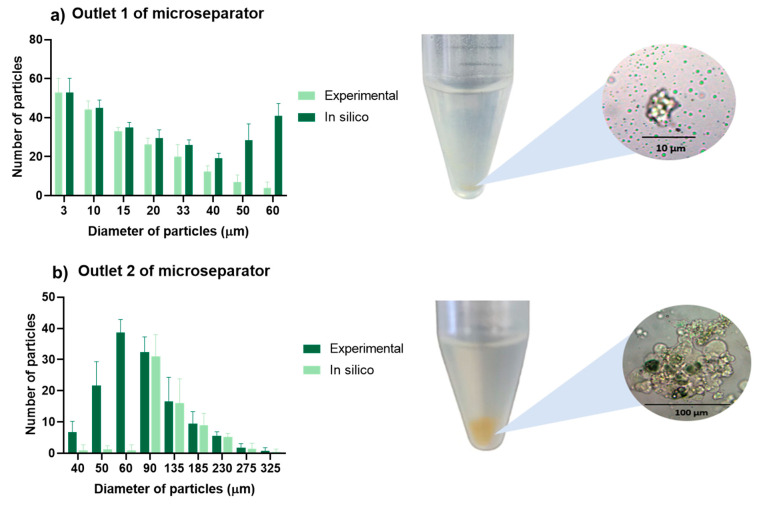
Chitosan microparticle separation performance in the Zweifach–Fung microfluidic device. (**a**) Distribution of particles at outlet 1, optimized for collecting particles under 40 μm; (**b**) particle distribution at outlet 2, designated for particles over 40 μm.

Utilizing the chitosan microparticle separation protocol developed for and applied in the evaluation of a prior device [64], we conducted a series of tests to rigorously analyze the device’s performance. The results are illustrated in Figure 6, where panel (a) details the particle distribution at outlet 1—intended for smaller particles—while panel (b) displays the distribution at outlet 2, expected to segregate larger particles. This dual-outlet analysis provided a nuanced understanding of the device’s effectiveness across a range of particle sizes.

Under the optimal flow conditions identified in Figure 5, our comprehensive evaluation demonstrated exceptional efficiency in separating particles smaller than 40 μm, predominantly exiting through outlet 1. However, a significant deviation was observed with particles in the 50 to 60 μm range. The experimental recall rates here fell substantially short of the simulation predictions.

We hypothesize that this disparity may be due to partial blockages within the microchannels, a consequence of employing a low-cost laser ablation manufacturing process on PMMA. As illustrated in Figure 4 and corroborated by the literature, laser ablation, ethanol degradation, and the constriction effect generated during bonding results in a reduction in the microchannel width from 49.40 μm ± 9.16 μm to 44.85 μm ± 8.27 μm, corresponding to a reduction of 4.55 μm in channel length. We associated this reduction in microchannel lengths with obstructions that impede the transport of larger particles, thereby diminishing the efficacy of the ZF separation channels for this particle size range.

To establish a baseline for comparison with prior research, we adhered to the same classification criteria as in our previous studies [68]. Particles with diameters less than 40 μm were deemed positive, while those exceeding 40 μm were classified as negative. This standardized metric ensured methodological consistency and enabled reliable assessment of the device’s performance.

Our calculations revealed that the device achieved an average accuracy of 94.4% ± 3% with a recall of 96.5% ± 3%. The precision stood at 94.5% ± 3%, and the F1 score—a measure that combines precision and recall—was 95.4% ± 3%. These robust figures underscore the exceptional capability of the ZF system to not only recall particles with high efficiency but also to do so with remarkable precision and reliability.

The performance metrics of the eleven-channel microfluidic device developed in this study were compared to other microfluidic systems documented in the literature, as detailed in Table 2. Our device manufactured using CO_2_ laser ablation in PMMA present the possibility of scaling up production at a cost of less than USD 0.90 per chip, and the manufacturing process takes only 15 min, which are important advantages for the device compared with the other microfluidic devices presented in the literature.

The efficiency of our device demonstrates favorable results when juxtaposed with other microfluidic technologies utilizing the ZF approach produced by alternative manufacturing processes. Our device shows comparable outcomes to other methodologies employed in similar microfluidic systems. Specifically, the precision of the ZF system maintained a high level of accuracy, aligning closely with the 96% precision reported in our previous study [68], indicating consistent reliability. Notably, there was a substantial improvement in recall performance, which increased from 77.62% in the earlier device to a significantly higher value in the current system. This enhancement in recall is further corroborated by the F1 score, which exhibited a notable rise from 79.70% to the present figure, underscoring the advancements achieved in the design and operational parameters of the current ZF system.

These results highlight the effectiveness of the improvements made to the ZF system and suggest that the advancements in this microfluidic device contribute to its superior performance compared to previously developed systems.

### 3.5. SWOT Analysis (Strengths, Weaknesses, Opportunities, and Threats)

**Figure 7:**
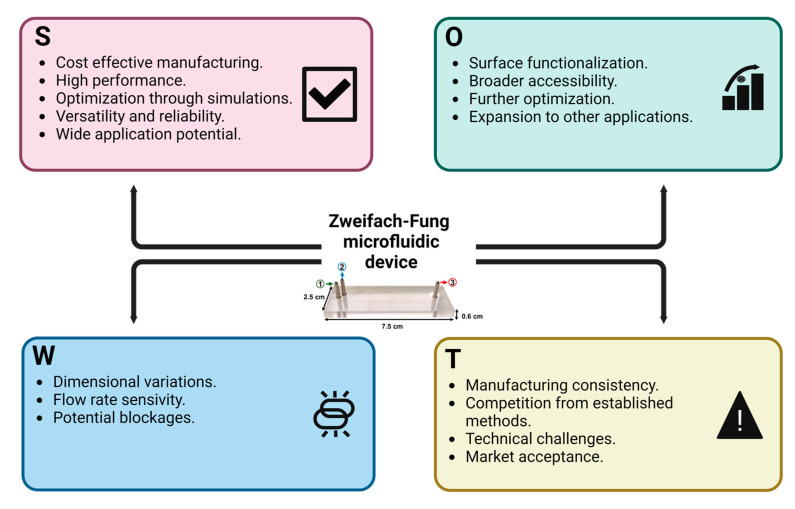
SWOT Analysis of the Zweifach–Fung Microfluidic Device. (S) Strengths, (W) weaknesses, (O) opportunities, and (T) threats.

The novel Zweifach–Fung microfluidic device presented in this article demonstrates several key strengths, weaknesses, opportunities, and threats (SWOT) that merit consideration, as shown in Figure 7 [19]. One of the primary strengths of this device is its cost-effective manufacturing process. Utilizing a low-cost laser ablation method in PMMA reduces production costs to less than USD 0.90 and eliminates the need for cleanroom facilities. This affordability does not compromise performance, as the device achieves an overall accuracy of 94.4% ± 3%, indicating exceptional precision and recall. The use of Lagrangian computational simulations to optimize the eleven-channel Zweifach–Fung configuration further ensures high efficiency in particle separation. Additionally, the device maintains steady operational conditions, providing reliable and reproducible results that contrast favorably with traditional methods. Its versatility and reliability make it applicable across various fields where separate large amount of microparticles or microcapsules are necessary, including fields such as the food, pharmaceutical, oil and gas, and chemical industries, thereby promoting and accelerating the process and efficiency [77,78,79,80,81,82].

However, the microfluidic device is not without its weaknesses. The manufacturing process introduces minor dimensional errors, reducing channel widths by approximately 5 μm post-bonding, which could affect the performance. Additionally, the device’s efficiency is highly sensitive to flow rates. Higher total flow rates significantly reduce separation efficiency, with a marked drop-in recall rate observed at increased flow rates. Furthermore, potential blockages due to sedimentation pose a challenge, potentially hindering consistent performance.

Despite these weaknesses, there are significant opportunities for improvement and broader applications. Future iterations of the device could incorporate surface functionalization techniques to minimize particle adhesion, thereby enhancing operational reliability [83,84,85,86]. The cost-effective nature of the device increases the accessibility of microfluidic technology, potentially leading to wider adoption and further innovation in the field. Additional research and development efforts could optimize the device’s design and operational parameters, improving efficiency and reducing dimensional variability.

Nonetheless, the device faces several threats that could influence its widespread adoption. The inherent variability in the low-cost manufacturing process might lead to inconsistencies in the device performance, affecting reliability. Competition from established microfluidic fabrication methods could limit the adoption of this new approach, particularly in industries where traditional methods are preferred. Continued technical challenges in ensuring precise control over channel dimensions and avoiding blockages could further impede widespread use. Lastly, market acceptance remains a potential threat; despite its advantages, the device may encounter resistance from industries accustomed to conventional methods, necessitating significant effort to demonstrate its efficacy and reliability.

## 4. Conclusions

This study conclusively demonstrates that an eleven-channel Zweifach–Fung microfluidic device configuration is essential for optimal particle recall, achieving 100% retrieval of particles conforming to a normal distribution with a mean diameter of 20 μm and a standard deviation of 6.7 μm. Through rigorous Lagrangian computational simulations, the research established 2 mL/h as the ideal operational flow rate, significantly enhancing the precision of particle separation to 94.4% ± 3%, a marked improvement from the previously recorded precision rate of 77.6% [64].

The study demonstrated a reduction in channels length by 4.55 μm, which was associated with the low-cost manufacturing processes employed, specifically laser ablation in the PMMA and the use of ethanol in the assembly. These processes likely alter the microchannel dimensions, subsequently impeding particle passage. This finding is crucial for the manufacturing of microfluidic devices with channels smaller than 100 μm using this low-cost approach. Therefore, it is recommended that future designs account for this reduction by developing microchannels 4.55 μm longer than initially planned to accommodate the manufacturing technique’s inherent variation.

Moreover, despite the exceptional separation efficiency of the eleven-channel Zweifach–Fung device under optimal flow conditions, issues such as sedimentation leading to potential blockages were observed. Future iterations of the device design could benefit from integrating surface functionalization techniques to minimize particle adhesion, thus enhancing operational reliability while maintaining the cost-effectiveness of the fabrication process that eschews the need for cleanroom facilities. In sum, this research represents a substantial leap forward in the field of microfluidic separation technologies. The study not only underscores the capability of passive microfluidic devices to achieve high precision and reliability but also illustrates the feasibility of scaling such technologies through cost-effective manufacturing approaches. The Zweifach–Fung microfluidic device, with its refined design and optimized operational parameters, consistently delivered high accuracy, averaging 98% under ideal conditions, setting a new benchmark for performance in the realm of microfluidic applications.

## Figures and Tables

**Table 1:** Materials for the Manufacturing of the Microfluidic Device.

Material	Quantity	Cost per Unit (USD)	Total Cost (USD)
Microfluidic Connectors	3 units	USD 0.05	USD 0.15
PMMA Sheets (7.5 cm × 2.5 cm)	2 units	USD 0.26	USD 0.51
	1 mL	-	USD 0.071
	5 mL	-	USD 0.02
	1 mL	-	USD 0.069
**Total cost**			**USD 0.82**

**Table 2:** Comparative Analysis of Microfluidic Devices in the Literature.

Literature	Our Work	[26]	[71]	[72]	[64]	[73]	[57]	[74]	[75]	[76]
**Channel Geometry**	T-shaped	U and W-shaped	Curved	T-shaped	Wave	Spiral-shaped	T-shaped	V complex-shaped	T and Y-shaped	T-shaped
**Material**	PMMA	PMMA	PDMS	PDMS	PMMA	PDMS	PMMA	PMMA	PDMS	PMMA
**Fabrication Technique**	CO_2_ laser	CO_2_ laser	Soft lithography	Photolithography	CO_2_ laser	Soft lithography	Soft lithography	Photolithography	Lithography	Lithography
**Depth**	60 µm	162–210 µm	40 µm	50 µm	-	-	-	20 µm	10 µm	20 µm
**Channel Width**	60 µm	180–227 µm	100–200 µm	300–700 µm	300 µm	15–40 µm	10–20 µm	9.6–15 µm	100 µm	
**Cross Section**	Gaussian	Gaussian	Gaussian	Rectangular	Gaussian	Rectangular	Rectangular	Gaussian	Rectangular	Rectangular
**Flow Rate**	2 mL/h	42 mL/h	-	-	23 mL/h	0.18–0.42 and 1.5–6 mL/h	6 mL/h	1–10 mL/h	0.01 mL/h	10 mL/h
**Separation Technique**	Zweifach–Fung effect	Hydrodynamic force	Centrifugal force, Coriolis force	Zweifach–Fung effect	Zweifach–Fung effect	Dean drag force	Zweifach–Fung effect	Plasma skimming effect	Zweifach–Fung effect	Zweifach–Fung effect
**Cost per Chip**	<USD 0.90	<USD 1.00	<USD 1.00	>USD 1.00	<USD 2.00	>USD 1.00	>USD 1.00	>USD 1.00	>USD 1.00	>USD 1.00
**Fabrication Time**	15 min	-	-	>1 h	15 min	>2 h	-	-	-	<1 h
**Separation Efficiency**	94%	92–96%	99%	99.70%	96.14%	8–13%	66.6%	65.1–100%	0.25	0.4
**Particle Size**	<40 µm	RBC size	RBC size	RBC size	15–40 µm	RBC size	RBC size	RBC size	8–16 µm	RBC size

## Data Availability

The original contributions presented in the study are included in the article/Supplementary Materials; further inquiries can be directed to the corresponding author/s.

## Supplementary Materials

The following supporting information can be downloaded at: https://www.mdpi.com/article/10.3390/mi15070932/s1, Figure S1: Mesh convergence analysis; Figure S2: Close-up image of one of the fabricated microfluidic devices; Figure S3: Experimental setup employing the syringe infusion pump; Table S1: Properties of the simulated particles [64,87]; Table S2: Number of domain elements and boundary elements of the simulated microfluidics devices.
