# Supplementary material for: Zweifach–Fung Microfluidic Device for Efficient Microparticle Separation: Cost-Effective Fabrication Using CO2 Laser-Ablated PMMA
Source: Micromachines (Basel). 2024 Jul 22;15(7):932. doi: 10.3390/mi15070932 (PMC11278838; doi:10.3390/mi15070932)
Supplement: Supplementary file 1 [file micromachines-15-00932-s001.zip › micromachines-3087053-supplementary.pdf]

micromachines

SUPPLEMENTARY MATERIAL

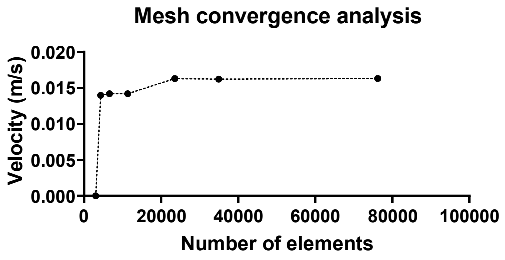

Figure S1. Mesh convergence analysis.

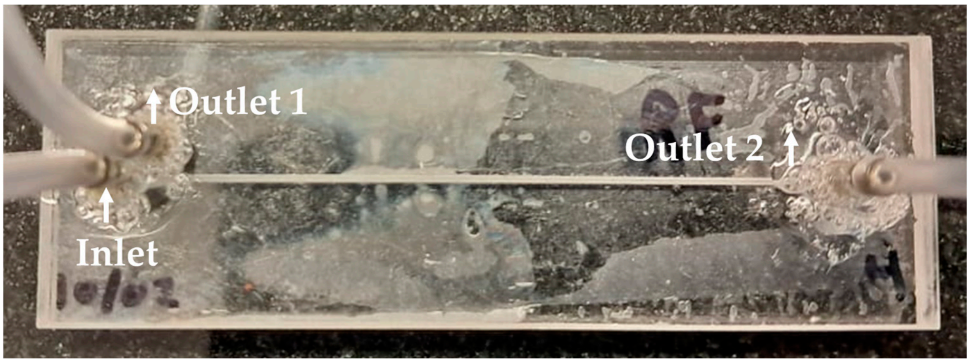

Figure S2. Close-up image of one of the fabricated microfluidic devices, showing the inlet and two outlets (Outlet 1 and Outlet 2).

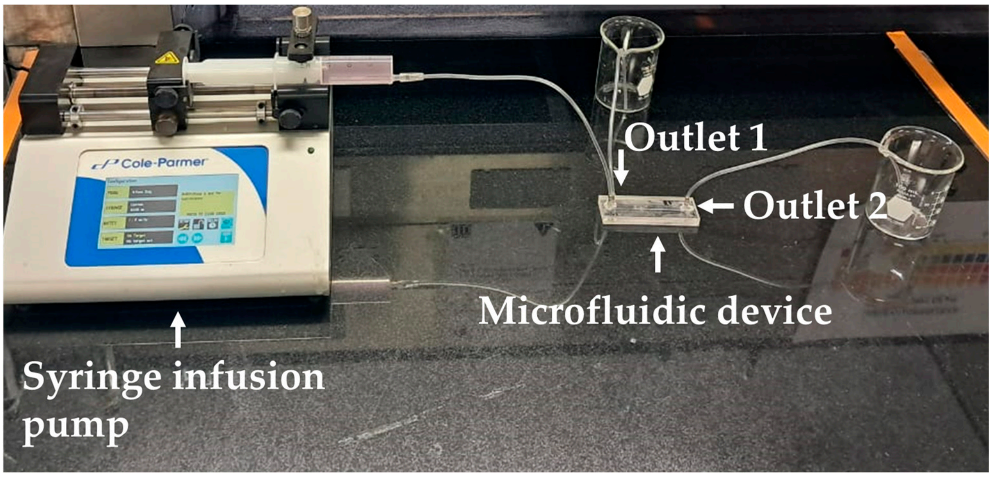

Figure S3. Experimental setup employing the syringe infusion pump. The setup includes the syringe infusion pump connected to the microfluidic device, with two distinct outlets, Outlet 1 and Outlet 2, as shown in the image.

Table S1. Properties of the simulated particles. References of the parameters used in literature [1], [2.]

Simulation Scenario	Particle Type	Density (kg/m³)	Results
Passive Separator Optimization	General Simulation	1050	Figure 4
Analysis of total flow rate passive separator	Polystyrene Microparticles	1050	Figure 5
	Chitosan Microparticles	1400	Figure 6

Analysis of total flow rate pas	

Table S2. Number of Domain elements and Boundary elements of the simulated microfluidics devices.

Microfluidics devices	Domain elements	Boundary elements
One-channel Zweifach-Fung	22.607	2.568
Two-channel Zweifach-Fung	23.457	2.668
Three-channel Zweifach-Fung	24.047	2.740
Four-channel Zweifach-Fung	24.534	2.795
Five-channel Zweifach-Fung	24.928	2.845
Six-channel Zweifach-Fung	25.224	2.889
Seven-channel Zweifach-Fung	25.596	2.929
Eight-channel Zweifach-Fung	23.613	2.948
Nine-channel Zweifach-Fung	23.819	2.968
Ten-channel Zweifach-Fung	23.534	2.945
Eleven-channel Zweifach-Fung	23.573	2.946
Twelve-channel Zweifach-Fung	23.377	2.916

References

1. C. E. Torres et al., "Microfluidic Synthesis and Purification of Magnetoliposomes for Potential Applications in the Gastrointestinal Delivery of Difficult-to-Transport Drugs," Pharmaceutics, vol. 14, no. 2, p. 315, Jan. 2022, doi: 10.3390/pharmaceutics14020315.
2. C. F. Rodríguez et al., "Low-cost inertial microfluidic device for microparticle separation: A laser-Ablated PMMA lab-on-a-chip approach without a cleanroom," HardwareX, vol. 16, p. e00493, Dec. 2023, doi: 10.1016/j.ohx.2023.e00493.

Disclaimer/Publisher's Note: The statements, opinions and data contained in all publications are solely those of the individual author(s) and contributor(s) and not of MDPI and/or the editor(s). MDPI and/or the editor(s) disclaim responsibility for any injury to people or property resulting from any ideas, methods, instructions or products referred to in the content.
